# Mechanical constraints on cell plasticity: insights from the olfactory epithelium

**DOI:** 10.3389/fcell.2026.1841670

**Published:** 2026-08-14

**Authors:** Raphaël Aguillon, Pascale Dufourcq, Julie Batut

**Affiliations:** 1 Laboratoire de Photobiologie et Physiologie des Plastes et des Microalgues, CNRS, Sorbonne Université, Institut de Biologie Physico-Chimique, Paris, France; 2 University of Toulouse, CNRS, Centre de Biologie Integrative CBI, Toulouse, France

**Keywords:** cell fate, mechanical constraints, mouse, olfactory epithelium, plasticity, zebrafish

## Abstract

The olfactory epithelium (OE) is a unique model to study how mechanical forces and tissue architecture regulate stem cell plasticity. In vertebrate, its lifelong neurogenesis and regenerative capacity depend on horizontal basal cells and globose basal cells, whose plasticity is gated by niche architecture and morphogenetic cues. While molecular pathways in OE development and regeneration are well-characterized, the role of physical forces, from basal niche stiffness to lamellar folding, remains unexplored. This review integrates single-cell analyses, developmental studies, and mechanobiology to propose that the unique structure of the OE (pseudostratified epithelium, spatial compartmentalization) and conserved pathways (YAP, CXCR4, retinoic acid) coordinate plasticity across ontogeny. We discuss how disrupted mechanoregulation might drive olfactory deficits in aging, infection (COVID-19), congenital disorders (Kallmann syndrome), cancer, and outline key questions and processes to decode the mechanobiological rules governing stem cell and progenitor plasticity. A table summarising the tools available at the cellular and tissue levels for manipulating forces and visualising their effects is provided.

## 1 Introduction

How much of our decisions are constrained by the environment? The capacity of stem cells to generate specific cell types at precise times and locations result from intrinsic programs interacting with their environment (Wells and Watt, 2018). The neuronal OE stands out due to its direct exposure to the external world: in adult, olfactory sensory neurons (OSNs) extend ciliated dendrites into the nasal cavity to detect odorants, while their axons project to the olfactory bulb to relay sensory information. This dual interface with both the environment and the central nervous system demands lifelong neurogenesis to maintain function despite constant exposure to damage (Graziadei and Graziadei, 1979; Graziadei and Monti Graziadei, 1979). During embryogenesis, the OE originates from the olfactory placode (OP), a transient ectodermal structure that gives rise to a pseudostratified neuroepithelium composed by several types including OSNs, support and stem cells (Aguillon et al., 2016). The unique developmental trajectory of the OP, marked by morphogenetic movements and interactions with neural crest cells (Breau et al., 2017) establishes the foundation for the adult OE with remarkable regenerative capacity.

**FIGURE 1:**
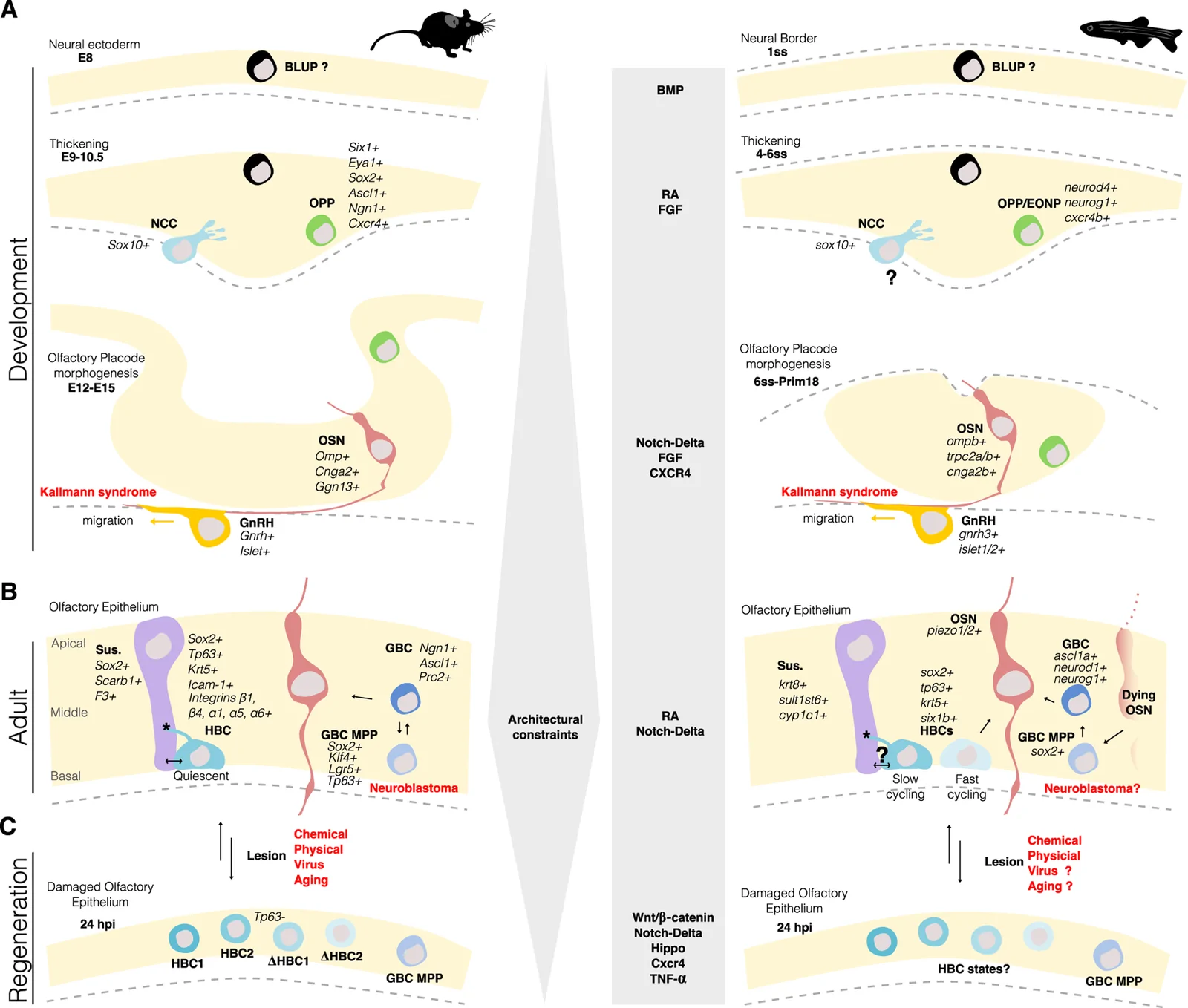
Comparative development, architecture, and regeneration of the olfactory epithelium in mouse and zebrafish. **(A)** Development: Left, Mouse: putative BLUPs (black) emerge in the neural ectoderm (E8), followed by thickening (E9-10.5) of olfactory placode progenitors (OPP, green) and neural crest cells (NCC, blue). Olfactory placode morphogenesis (E12-E15) results in OSNs (red) and GnRH neuron migration (yellow). Right, Zebrafish: putative BLUPs (black) potentially emerge at the neural border (1ss), with thickening (4–6ss) of OPP/EONP (green) and NCC (blue). Olfactory placode morphogenesis (6ss–Prim18) with OSNs (red) and GnRH neurons (yellow). Kallmann syndrome can arise from defects in GnRH migration in both species. In grey dotted line, the underlying and overlying (only zebrafish) constraining layers. **(B)** Adult Olfactory Epithelium: Left, Mouse: Apical sustentacular cells (purple), middle-layer OSNs (red), and basal quiescent HBCs (light blue) and GBCs (dark and light blue). GBCs can adopt either a multipotent progenitor (MPP) or a neuronal progenitor (GBC) state. Right, Zebrafish: Apical sustentacular cells (purple), middle-layer OSNs (red), and basal HBCs (turquoise and light turquoise) with fast- and slow-cycling populations, and GBCs (dark and light blue) that also adopt a putative MPP or neuronal progenitor state. In grey dotted line, the underlying constraining layers. **(C)** Regeneration: Left, Mouse: Lesion (in red, Chemical: Ethidium Bromide, Methimazole, Triton-X100. Physical: Axotomy, Olfactory bulbectomy. Viral: SARS-COV-2. Aging) induces activation of HBCs (HBC1, HBC2, ΔHBC1, ΔHBC2) and GBC MPPs at 24 hpi, hour-post-injury. Right, Zebrafish: Lesion induces proliferation of HBCs and GBC MPPs at 24 hpi. In grey dotted line, the underlying constraining layers. Figure center: The grey diamond represents the architectural constraints (Forces) dynamic across development, adult and regeneration, while the grey rectangular display main signaling pathways involved in each depicted process.

The OE provides a rare opportunity to dissect how the environment regulates plasticity across ontogeny, from development to adult regeneration. Here, stem cells (e.g., horizontal basal cells, HBCs) self-renew and generate progenitors (e.g., globose basal cells, GBCs) which differentiate into the diverse cell types required for odor detection and epithelial integrity (Figure1). Early models attributed OE regeneration to diffusible signals originating from dying cells (Calof et al., 1996; Imayoshi et al., 2015) and inflammatory cues (Choi and Goldstein, 2018; Kern et al., 2004) that override stem cells quiescence. However, emerging evidence implicates tissue-level architecture (Table1: Key terms definition) as a regulator: intact epithelium suppresses stem cell activation, while structural disruption permits cell plasticity (Schwob et al., 2017; Kang et al., 2025). This mechanically gated model, where plasticity is transiently unlocked when tissue organization loosens and actively suppressed upon restoration, positions the OE as an ideal system to explore how forces, niche geometry, and extracellular matrix (ECM) interactions coordinate stem cell behavior. Similarly, during development, olfactory cell identity emerges through morphogenetic movements (Breau et al., 2017), implying that fate specification and spatial reorganization are co-regulated by mechanical cues (Poncelet and Shimeld, 2020).

**TABLE 1:** Key terms definition and cell-tissue-scale tools with references.

Key terms	Definition	
Architecture	The spatial and physical organization of a tissue, including cellular arrangement, extracellular matrix structure, and mechanical properties	
Mechanical constraints	Physical forces (e.g., tension, compression, stiffness, or spatial confinement) that regulate cellular processes	
Regeneration	Restoration of tissue structure and function after damage, driven by stem/progenitor cell activation, lineage reprogramming, and tissue remodeling to repair or replace lost cells	
Plasticity	Competence of a cell population to modify its lineage or fate potential (e.g., expand, restrict, or switch fates) in response to environmental cues during development and regeneration	

Cell-scale tools	Force type (Visualise/Manipulate)	References
FliptR: Fluorescent lipid tension Reporter	Membrane tension (stretching)	Colom et al. (2018)
VE-cadherin FRET	Cell-cell tension (intercellular forces)	Lagendijk et al. (2017)
Actin FRET sensor	Actin fiber tension (cytoskeletal stress)	Yamashita et al. (2016)
AFM	Cell elasticity (stiffness, indentation)	Krieg et al. (2008); Marsal et al. (2017)
Opto-RhoGEF	Cell contractility (RhoA activation)	Izquierdo et al. (2018)
Opto-RhoA	Actin polymerization (RhoA activation)	Berlew et al. (2021)
Opto-MYPT	Inhibition of membrane-actin activity (myosin regulation)	Yamamoto et al. (2021)
Tissue-scale tools		
Laser ablation + E-cadherin-GFP	Tissue tension (recoil velocity after ablation)	Kong et al. (2019)
GPI-RFP; Tau-mCherry; Myl12.1-GFP	Tension inference (cell shape, mitotic spindle, actin fibers)	Campinho et al. (2013)
H2B-RFP	Velocity field and strain mapping (tissue deformation)	Bhattacharya et al. (2021)
Laser ablation + Actine-Myl12.1-GFP	Actin network forces (recoil dynamics)	Smutny et al. (2015)
2-photon laser ablation	Tensile stress (cell protrusion/retraction dynamics)	Marshall et al. (2022)
Fluorescent lipid droplets	Tissue tension (geometry-based inference)	Campàs et al. (2014)
Ferrofluid droplets	Intra/extracellular tension (deformation dynamics)	Serwane et al. (2017)
Lipid droplet osmotic probe	Tissue-scale tension (osmolarity changes)	Vian et al. (2023)
Brillouin microscopy	Tissue mechanical properties (stiffness, elasticity)	Bevilacqua et al. (2019)
Optical elastography	Tissue mechanical properties (spatially resolved elasticity)	Tomizawa et al. (2022)
ForSys: A python-based open-source software to infer mechanical stress in tissues	Static/dynamic tension (force fields)	Borges et al. (2025)
Capillary aspiration	Applies traction forces (mechanical perturbation)	Dassow and Davidson (2009)
Agarose shape	Applies geometric constraints (tissue deformation)	Behrndt et al. (2012)

This interplay between intrinsic programs and environmental constraints raises a fundamental question: to what extent do mechanical forces and architectural cues instruct, permit, or restrict stem cell plasticity in the OE? While molecular pathways governing OE development and regeneration have been extensively characterized, the role of physical forces, from basal niche mechanics to tissue-scale morphogenetic stresses, remains largely unexplored. By integrating single-cell analyses, classical developmental studies and detailed cellular interactions during OE development and regeneration across species, this review examines how the unique OE structure provides a model system to decode the mechanobiological rules governing cell plasticity**.**

## 2 Plasticity within the OE

### 2.1 Plasticity seems to operate at different cell levels within the OE

Plasticity in the OE appears to be a property emerging at both cellular and tissular scales. Neurogenesis in the adult mouse OE is spatially compartmentalized into active and quiescent zones, indicating heterogeneity within the regenerative populations based on location (Graziadei and Monti Graziadei, 1979; Graziadei and Graziadei, 1979). The OE is a pseudoepithelium with cell types distributed along the apico-basal axis. Quiescent HBCs, the olfactory stem cells, anchor themselves to the basal layer, poised to respond to injury or regenerative cues (Figure 1B, Adult, Basal). Just above, GBCs occupy a suprabasal niche, actively proliferating to sustain the continuous turnover of olfactory sensory neurons and other epithelial lineages in homeostatic conditions (Figure 1B, Adult, Basal and Middle). Moving further apically, immature and mature OSNs extend their dendrites toward the epithelial surface (Figure 1B, Adult, Middle). Interspersed among these neurons, sustentacular cells span the apical region, providing metabolic support and maintaining the structural integrity of the epithelium (Figure 1B, Adult, Apical). Finally, microvillar cells and Bowman’s gland (not shown on the figure) contribute to the apical environment, facilitating odorant solubility and clearance.

While plasticity in the olfactory system was attributed to the intrinsic multipotency of individual basal progenitors and stem cells, recent clonal and single-cell analyses in mouse have revealed that both HBCs and GBCs comprise heterogeneous populations, challenging traditional definition of plasticity. After lesion in mouse, HBCs can generate all OE cell types *in vitro* (Carter et al., 2004) and *in vivo* (Gadye et al., 2017). Recent single-cell analyses in mouse have identified multiple HBCs states (resting HBC1/HBC2, and activated ΔHBC1/ΔHBC2) that emerged as soon as 24 hour-post-injury (24 hpi) (Gadye et al., 2017). Trajectory analyses further demonstrated that differentiation paths require transitions through specific combinations of these states (Gadye et al., 2017; Fletcher et al., 2017). Intriguingly, aging and neurogenic depletion in humans and mouse preserve HBCs but bias their differentiation toward sustentacular cells, leading to olfactory decline. This suggests loss or dysfunction of neurogenic HBC subpopulations, compromising OE regeneration (Child et al., 2018). In adult zebrafish, HBCs display even greater heterogeneity, both spatially and functionally (Figure 1B, zebrafish, Adult). Unlike their largely quiescent mammalian counterparts, zebrafish HBCs are constitutively active, with ∼70% dividing within a week under homeostasis (Kocagöz et al., 2022). This activity is spatially patterned within the OE: HBCs at the interlamellar curves (ILC) and sensory/non-sensory borders (SNS**)** divide more frequently (∼8.7-day cycle), while those in the core sensory OE exhibit slower cycling (∼15.7-day cycle) (Figure 1B, zebrafish, Adult). Upon injury, the basal stem cell compartment shows active proliferation compared to intact OE as early as 24 hpi (Kocagöz et al., 2022). Single-cell RNA-seq (scRNAseq) of intact OE further reveals a complex transcriptional landscape for zebrafish HBCs (Takaoka et al., 2025), highlighting distinct regulatory mechanisms of plasticity in zebrafish compared to mammals.

GBCs, which sustain most OE lineages under homeostatic conditions, exhibit a heterogenous molecular identity in mammals. In intact mouse OE, GBCs are characterized by partially overlapping expression of *Ngn1*, *Ascl1, Prc2 and Tp63*, *Lgr5*, *Sox2* and *Klf4*, reflecting their function as neuronal progenitor or multipotent progenitor (MPP) respectively (Figure 1B, Adult, Basal and Middle) (Ko et al., 2023; Chen et al., 2014; Lin et al., 2017; Fletcher et al., 2017). ScRNA-seq analyses after injury have resolved murine GBCs into discrete subpopulations, revealing a mixed landscape of progenitors and stem-like cells with distinct regenerative capacities (Fletcher et al., 2017; Gadye et al., 2017). These subpopulations contribute to homeostatic neurogenesis, generating OSNs and sustaining OE cellular turnover. In adult zebrafish, this heterogeneity is both conserved and expanded, with recent scRNA-seq studies under homeostatic conditions (Takaoka et al., 2025) refining our understanding of GBC diversity. Zebrafish GBCs (expressing *neurod1, neurog1* and *ascl1*) are spatially restricted to the ILC and SNS regions, where they drive maintenance neurogenesis (Bayramli et al., 2017). Unlike their mammalian counterparts, zebrafish GBCs seem to exhibit a more streamlined transcriptional profile, clustering tightly in UMAP analyses (Takaoka et al., 2025).

### 2.2 Plasticity is a developmental feature of the OE enabling cell types establishment

Plasticity is also a hallmark of olfactory developmental cells, reflecting a conserved strategy for generating cellular diversity. Lineage studies in chicken and mouse revealed that the OE cell type diversity arises from two distinct developmental lineages: the OP progenitors (OPP) and NCC (Figure 1A, Development) (Katoh et al., 2011; Forni et al., 2011). Notably, in the adult mouse OE, GBCs express molecular markers (*Sox2, Ascl1 and Ngn1*) shared with OPP during morphogenesis stage (*Six1, Eya1, Sox2, Ascl1 and Ngn1*) (Packard et al., 2011; Cau et al., 1997; Panaliappan et al., 2018) suggesting a developmental continuity (Figures 1A,B, mouse, Development and Adult). Single-cell analyses spanning OP development in chick enriched this dual origin: Border-Located Undecided Progenitors (BLUPs) co-exist with, and co-express markers of, both OPP and NCC populations (Thiery et al., 2023). However, their existence in other vertebrates, including mammals and zebrafish, remains to be confirmed (Figure 1A, Development). The discovery of Pre-Migratory NCC in amphioxus, which also express genes from both lineages, suggests the existence of a common ancestral population underlying these vertebrate innovations (Markos et al., 2024). Notably, the ongoing debate about NCC contribution to the OE in zebrafish likely stems from the evolutionary, developmental, and physical proximity of these two progenitor populations (Figure 1A, zebrafish, Development) (Whitlock et al., 2003; Saxena et al., 2013; Aguillon et al., 2018). This proximity may have led to transient or context-dependent interactions during development, which could explain conflicting results across studies. Subsequent single-cell analyses of the olfactory territory over the course of zebrafish development are necessary to further characterize, and disentangle, OE cell types and their developmental origins. In zebrafish, *neurog1* expressing OPP cells are also called Early Olfactory Neurons Progenitors (EONP) (Figure 1A, zebrafish, Development) (Madelaine et al., 2011; Aguillon et al., 2020; Zilliox et al., 2025).

Plasticity is a core feature of the OE, enabling progenitor cells and stem cell populations to dynamically adjust their fate in response to developmental, regenerative, and environmental demands. In adults, heterogeneity within HBCs and GBCs underscores the role of plasticity in adaptation and repair, while developmental studies reveal its evolutionary roots in dual-lineage origins and undecided progenitor states.

### 2.3 Factors contributing to plasticity closure during OE development

Plasticity closure in the OE is regulated by the coordinated interaction of transcriptional programs and morphogenetic cues, which collectively restrict progenitor potential as development progresses. In mouse, chick and *Xenopus* the OPP (Figure 1A, Development) become determined, hallmark of a closing plasticity, during the invagination which is the first morphogenetic event of the OP (Chen et al., 2009; Bhattacharyya and Bronner-Fraser, 2008; Cornesse et al., 2005). Notably, single-cell analyses during chick OP development reveal that the BLUP population undergoes fate restriction, splitting into distinct OPP and NCC lineages (Thiery et al., 2023). This bifurcation marks a critical transition in plasticity closure. Whether BLUPs exist in zebrafish and mouse during early OP development remains unknown, and identifying these cells could provide insights into conserved mechanisms of plasticity closure across vertebrates (Figure 1A, Development). As development proceeds, key transcriptional regulators such as *Sox2* and *Six1/4* with growth factor such as Fibroblast growth factors (*Fgf8*), become essential for both OE neurogenesis and OP morphogenesis in chick and mouse (Figure 1A, Development) (Panaliappan et al., 2018; Kawauchi et al., 2005; Chen et al., 2009), revealing that differentiation and tissue shaping are co-regulated at the molecular level. In zebrafish, Neurog1 in the OP further illustrates this coordination, by directly controlling the expression of both the neurogenic Notch/Delta pathway and *cxr4b,* the chemokine receptor, regulating OP morphogenesis through EONP positioning (Madelaine et al., 2011; Madelaine and Blader, 2011; Aguillon et al., 2020).

Morphogenetic forces further refine OE structure through temporal shifts in mechanical cues. Early OPP respond to tractive forces from the eye primordium (10–26 somite stages, 10–26ss), mediated by the Extra Cellular Matrix, ECM (Monnot et al., 2022). Later, at primordium 12 stage (Prim-12), OSNs take an active role, using actomyosin-dependent contractility to pull overlying skin cells and form the nostril (Figure 1A, zebrafish, Development) (Baraban et al., 2023). This sequential process underscores how early external cues from the eye primordium set the stage for later OSN-driven morphogenetic events. Supporting this dynamic interplay, heterograft experiments in *Xenopus* demonstrate that transplanted OP (already undergoing morphogenesis) tissue can induce local morphogenetic changes in the diencephalon, with cells emigrating from the grafted tissue to integrate into host structures (Magrassi and Graziadei, 1985; Koo and Graziadei, 1995). While local forces, cell-cell-ECM interactions, and ECM composition are beginning to be functionally addressed during OE morphogenesis (Table1: Cell-Tissue-scale tools), particularly in zebrafish, thanks to its imaging advantages (Tignard et al., 2024), the initiation of morphogenesis and its interplay with plasticity regulation remain poorly understood.

Critically, the migration and morphogenesis of OP cells not only shape the olfactory epithelium but also create a structured environment essential for other cell populations, such as *GnRH* neurons, to migrate and differentiate properly (Figure 1A, Development). The GnRH case exemplifies this coordination: although originating early from adjacent OP territories, fate-mapping and trajectory analyses reveal that GnRH progenitors converge with the forming olfactory field during placode assembly (Whitlock and Westerfield, 2000; Aguillon et al., 2018), benefiting from local FGF and Retinoic acid (RA) signaling to be specified (Sabado et al., 2012). Their development thus coincides with integration into a spatially patterned and mechanically structured OP environment, highlighting how plasticity closure and tissue stabilization enable the precise assembly of functional neural circuits.

Together, these mechanisms illustrate how plasticity closure is a coordinated, multi-scale transition, from molecular fate restriction to tissue-level stabilization, essential for functional organ construction.

### 2.4 Molecular and mechanical features required for cell plasticity during loss of OE integrity

In adults, reopening plasticity in the OE follows a hierarchical response to tissue damage, where GBCs and HBCs are mobilized at distinct thresholds of injury. GBCs serve as first responders, exhibiting a highly sensitive activation threshold that enables them to replenish most OE lineages daily, even under moderate or physiologically routine damage. Their rapid and fine-tuned response ensures continuous homeostatic maintenance of the epithelium (Figure 1B, Adult). However, when tissue integrity is severely compromised HBCs are selectively recruited. As quiescent reserve stem cells, HBCs drive large-scale regeneration, restoring full epithelial architecture and function when GBC-mediated repair is insufficient (Figure 1C, Regeneration). At the core of this architectural regulation is *Tp63*, which acts as a central gatekeeper of horizontal basal cell in mouse and rats: its expression maintains HBC quiescence, while its downregulation is required for regenerative activation after lesion (Figure 1C, mouse, Adult and Regeneration) (Fletcher et al., 2017; Packard et al., 2011; Schnittke et al., 2015). In mouse OE, cell-cell contact plays a key role, as loss of sustentacular cells triggers HBC activation through *Tp63* downregulation in a Notch-dependent manner (Herrick et al., 2017; Louie et al., 2023). Structural mechanosensation further contributes to this response in rodents, with 70% of HBCs possessing primary cilia in direct contact with sustentacular cells (Joiner et al., 2015), potentially acting as damage sensors (Figure 1B, mouse, Adult). Mechanosensitive signaling is also involved, as evidenced by the upregulation of Yes-associated protein (YAP) in HBCs, mediated by sphingosine 1-phosphate, S1P/S1PR2 signaling, which drives lesion-responsive proliferation (Wu et al., 2022). While upstream mechanosensors such as Piezo ion channels that convert physical forces into biochemical signals remain to be characterized in mammalian OE stem cells, Piezo1/2 expression has been reported in adult zebrafish OE neuronal populations (Aragona et al., 2024), and Piezo2-mediated mechanotransduction has been demonstrated in olfactory bulb mitral cells of the OE-projecting circuit in adult rat (Figure 1B, zebrafish, Adult) (Salameh et al., 2024). Concurrently, β-catenin is upregulated in activated HBCs in both mouse and zebrafish (Fletcher et al., 2017; Kocagöz et al., 2022), linking mechanical disruption of niche contacts to transcriptional reprogramming and cell cycle re-entry. Finally, ECM-mechanosensitive adhesion completes the circuit: quiescent HBCs express integrins β1, β4, α1, α5, α6, and ICAM-1 above the laminin layer, all downregulated upon activation (Carter et al., 2004), suggesting that disruption of ECM contacts contributes to triggering plasticity in mammals (Figure 1C, mouse, Adult and Regeneration).

By contrast, GBCs respond to more localized cues, such as OSN-specific ablation. In adult zebrafish, their proliferation is triggered by dying OSNs via ATP/P2 signaling (Figure 1B, zebrafish, Adult) (Demirler et al., 2020). In adult mouse, under regenerative conditions, GBCs demonstrate remarkable plasticity by reverting to a MPP state, a transition marked by *polycomb repressive complex 2* (*Prc2*) downregulation, which relieves chromatin repression, and *Sox2*/*Klf4* upregulation (Yamanaka factors), enabling regeneration of all OE cell types (Figure 1B, mouse, Adult) (Ko et al., 2023). This epigenetic reprogramming suggests that PRC2-mediated chromatin compaction may act as a gatekeeper of GBC plasticity, analogous to the role of Tp63 in HBCs. Notably, mouse GBCs also express the chemokine receptor *Cxcr4*, a conserved marker shared with OP progenitors (Figures 1A,B, Development and Adult). The interplay between cell mechanics and cell fate is crucial in biological processes such as development, differentiation, and disease. Mechanical properties of the cellular environment, including stiffness and shear stress, significantly influence cell behavior, while changes in cell fate can alter these mechanics.

Cxcr4 regulates cell cycle exit and neurogenesis (Dietz et al., 2023; Senf et al., 2021) and has been implicated in OP development in both mouse and zebrafish (Aguillon et al., 2020; Toba et al., 2008; Memi et al., 2013; Zilliox et al., 2025; Miyasaka et al., 2007). Following lesion in adult mouse, Cxcr4 and its ligand Cxc12a are upregulated, correlating with increased GBC proliferation (Senf et al., 2021). Given the established role for Cxcr4 in cytoskeletal dynamics during neuronal circuit development (Shan et al., 2021) and its conserved expression in OP progenitors, this chemokine signaling axis presents a compelling entry point to investigate mechanoregulation of GBC plasticity. Most mechanistic studies have been conducted in mouse, but zebrafish development studies reveal that Cxcr4 is similarly critical for OP morphogenesis, suggesting evolutionary conservation of its function. Combined with unparalleled live-imaging capabilities of zebrafish, this model provides an ideal system to dissect how mechanical forces and Cxcr4 signaling interact to regulate progenitor behavior during both development and regeneration.

Thus, structural disruption (e.g., cell death, ECM detachment), mechanosensing (e.g., cilia, Yap), and epigenetic reprogramming (e.g., Prc2/Sox2) collectively enable OE stem cells to exit quiescence and restore tissue integrity.

## 3 Discussion

### 3.1 Failures in OE stem cell plasticity explain structural and functional olfactory deficits

Olfactory dysfunction affects 43% of COVID-19 patients (Bartheld et al., 2020), with most recovering, likely due to OE regeneration, as demonstrated in hamsters (Reyna et al., 2022). However, 5% remain anosmic after 6 months (Tan H. et al., 2023; Tan Z. et al., 2023, 20), pointing to a persistent regenerative defect. SARS-CoV-2 targets sustentacular cells via ACE2/TMPRSS2 (Bilinska et al., 2020; Bryche et al., 2020), causing OSN cilia loss and acute anosmia, while in hamsters, neutrophil/macrophage infiltration induces widespread cell death (Bourgon et al., 2022). Critically, it has been shown in human primary culture that TNF-α sustains TP63 expression in HBCs, enforcing quiescence and blocking neuronal regeneration (Oliva et al., 2022). In a murine model of olfactotropic viral infection, extensive tissue damage drives immune infiltration, ECM remodeling, and redirects surviving HBCs toward respiratory or osteogenic fates, demonstrating that severe niche disruption can override intrinsic stem cell programming (Tracey et al., 2025). While more severe than typical human post-COVID pathology, this supports the principle that maintaining niche architecture is critical for successful regeneration. Thus, persistent inflammation and disrupted niche architecture can each impair OE regeneration highlighting how cell plasticity and proper fate specification depend on intact niche signaling (Figure 1C, mouse, Regeneration).

Kallmann syndrome (incidence: 1 in 40,000–80,000) is a genetic disorder defined by anosmia and hypogonadotropic hypogonadism associated with gonadotropin-releasing hormone (GnRH) neurons development and function. During development, GnRH neurons transiently reside in the OP before migrating to the forebrain (Figure 1A, Development). Mutations in KAL1 (ASNOSMIN-1) or FGFR1 disrupt this process, impairing GnRH neurons migration and OP morphogenesis (Maione et al., 2018). These defects suggest that disrupted niche signaling, including FGF-dependent interactions between OP progenitors and GnRH neurons, compromises the establishment of functional olfactory circuits, highlighting how developmental plasticity closure can underlie congenital anosmia.

Olfactory neuroblastoma is a rare cancer (incidence: 4 per million) and remains understudied. However, recent studies in mouse and humans have traced its origin to GBCs, based on the expression of proneural factors (e.g., NeuroD1, ASCL1) and chromatin regulators such as EZH2 (PRC2 sub-unit) (Zunitch et al., 2023). These markers align with GBCs committed to the neuronal lineage, suggesting that neuroblastoma arises from a dysregulated plasticity state (Chen et al., 2009; Chen et al., 2014; Ko et al., 2023). Notably, EZH2, a plasticity inhibitor that maintains GBCs in a neuronal differentiation program (Lin et al., 2017), may favor tumor development by locking cells in a proliferative state, a hallmark of malignancy (Figure 1B, Adult).

In humans over 70 with presbyosmia (gradual degeneration of sense of smell due to ageing process), scRNAseq analysis detected upregulation of cytokine and stress pathways in HBCs. Primary culture experiments showed that TNF-α treatment increases TP63 expression, which closes plasticity and inhibits OE regeneration (Oliva et al., 2022). In aged mice, single-cell analysis revealed a loss of OSNs, accompanied by increased T-cells, neutrophils, and macrophages compared to younger mice, alongside respiratory metaplasia (replacement of OE with a non-sensory, ciliated, respiratory epithelium). Critically, T-cells inhibit HBC plasticity via IL-17α signaling, and blocking IL-17α reduces metaplasia, suggesting inflammation directly suppresses regeneration (Wang et al., 2025). Beyond producing inflammatory cytokines, immune cells such as tissue-resident macrophages and T cells may also function as architectural components of the regenerative niche. While immune cells in the OE are spatially organized within the lamina propria and can directly contact basal stem cells (Wellford and Ashley Moseman, 2024), whether immune-driven ECM remodeling influences mechanical niche cues to regulate HBC and GBC plasticity remains an open question. Another mechanism involving RA signaling has been pointed out as a key factor in respiratory metaplasia pathogenesis. RA acts as a conserved paracrine regulator of stem cell plasticity in both mouse and zebrafish: in mice, OSNs express CYP26B1 (RA catabolism) and sustentacular cells express RALDH1 (RA synthesis), locally modulating RA availability to control HBC behavior (Figure 1B, Adult). In respiratory metaplasia, RA availability is also involved in sustaining the non-neuronal niche, blocking restoration of the sensory epithelium (Håglin et al., 2020). In zebrafish embryo, RA produced by retinal cells expressing the enzyme Aldh1a2/3 from Prim-5 to Prim-16 influences olfactory progenitors by acting on their microenvironment, demonstrating a conserved niche process where daughter cells regulate stem cell plasticity via RA (Rajan et al., 2025).

Across these pathologies, COVID-19, Kallmann syndrome, neuroblastoma, and presbyosmia, failures in OE stem cell plasticity, whether due to chronic inflammation (TNF-α/IL-17α, with upregulation of TP63), dysregulated chromatin (EZH2), or a proposed altered RA signaling, lead to structural deficits (metaplasia, tumor growth) and functional loss (anosmia, malignancy). These mechanisms highlight how disrupted niche interactions block stem cells and the plasticity of progenitor cells, driving degeneration of the OE of congenital, age-related, infectious, or neoplastic origin.

### 3.2 Linking mechanic and cellular plasticity

Tissue functionality depends fundamentally on the precise spatiotemporal formation of cell types, a process where intrinsic stem/progenitor programs interact dynamically with their structural environment, a dimension often overshadowed by molecular determinants. The OE stands as an exceptional model to study this interplay, as decades of research have demonstrated that lineage establishment depends critically on surrounding tissue architecture. Crucially, evidence from both OE development and regeneration points to mechanical gating of progenitor/stem cell plasticity by the tissue microenvironment. The unique research landscape of the OE has evolved through two historically distinct fields: developmental formation and adult regeneration across multiple species. Recent advances, notably at the single-cell level, are now bridging these disciplines, creating an ontogenetic evo-devo framework to investigate mechanobiological principles with implications for both fundamental biology and regenerative medicine. Strikingly, despite decades of study in both fields, how mechanical forces regulate plasticity during OE development and regeneration, remains directly unaddressed. This gap presents an opportunity to redefine our understanding of the role of tissue architecture in stem cell regulation.

The diverse cell types of the adult OE originate from developmental progenitors whose trajectories are intimately linked to tissular morphogenesis. As development progresses, progenitor fates become increasingly stabilized and restricted, suggesting that morphogenetic forces may play a role in locking cellular identities (Figures A–C, Architectural constraints). However, while correlative evidence links morphogenesis to fate restriction, the causal role of mechanical forces in regulating progenitor plasticity remains unexplored in the OE. This stands in stark contrast to other developmental systems, where forces have been demonstrated as instructive cues: in *Drosophila*, compressive forces regulate β-catenin-dependent ventral and midgut identity (Desprat et al., 2008; Farge, 2003), while in *Xenopus*, hydrostatic pressure of the blastocoele modulates overlying neural crest competence through YAP/β-catenin suppression (Alasaadi et al., 2024). These findings are further supported by *in vitro* systems, where applied tension directs lineage decisions in human stem cells, ranging from stiffness-dependent differentiation in mesenchymal stem cells (MSCs) (Engler et al., 2006) to stretch-induced specification in human induced pluripotent stem cells (hiPSCs) (Kim et al., 2025) and geometric constraint-driven patterning in human pluripotent stem cells (hPSCs) (Xue et al., 2018; Muncie et al., 2020). Piezo ion channels, which convert physical forces into biochemical signals, have emerged as key mechanotransducers regulating stem cell quiescence and activation across multiple tissues, including gut, bone, epidermis, hair follicle, muscle in mice, and human neural stem cells, often through YAP/β-catenin pathways (Baghdadi et al., 2024; Xue et al., 2025; Foote et al., 2022; Xie et al., 2022; Pathak et al., 2014; Ma et al., 2022; Zhou et al., 2020). Given the upregulation of both YAP and β-catenin in activated HBCs, Piezo channels represent promising candidate mechanosensors that may similarly link mechanical forces to stem cell fate regulation throughout OE development and maintenance. Together, these studies establish mechanical forces as potent regulators of cell fate, raising the critical question of whether similar mechanisms govern plasticity restriction during OE development and regeneration.

The OE represents a unique pseudostratified neuroepithelium characterized by a two-layer spatial organization that underpins its functional and homeostatic capacities. Along the apico-basal axis, quiescent HBCs, the olfactory stem cells are preferentially localized to the basal layer, while GBCs, sustentacular cells, and OSNs occupy more apical positions with greater positional flexibility. This basal restriction of HBCs position raises fundamental questions about the relationship between spatial localization and stem cell identity: does the basal compartment actively contributes to stem cell regulation? Supporting this possibility, studies in other systems demonstrate that mechanical forces regulate stem cell positioning and population size. For example, during mouse incisor development, the actomyosin network critically regulates the size of Sox2 positive stem cell population (Hamadani and Myllymäki, 2026), while in early embryogenesis, differential contractility spatially segregates cells, separating the trophectoderm from the inner cell mass (Maître et al., 2012; Maître et al., 2015; Maître et al., 2016). While the basal layer of the OE provides potential mechanical interactions with the basement membrane and lamina propria that may regulate quiescence, the causal relationship between spatial localization and stem cell fate remains an open question in OE biology.

A second architectural feature of the OE is its lamellar folding, which generates spatial heterogeneity associated with functional differences within the epithelium. Although recent studies have begun to explore the role of forces in olfactory morphogenesis at the cellular scale (Monnot et al., 2022; Baraban et al., 2023), the tissue-level mechanical regulation of OE folding remains unknown. Comparative studies in chick and mouse gut development reveal striking parallels: epithelial lumen folding is driven by compressive stress from surrounding muscle layers (Shyer et al., 2013), with the formation of muscle layers themselves dependent on a combination of Hedgehog (Hh) signaling, Bone Morphogenetic Proteins (BMP) inhibition, and mechanical tension, first continuous, then cyclic (Huycke et al., 2019). Intriguingly, *in vitro* models using human stem cells recapitulate germ layer patterning when cultured in disk-shaped micropatterns with BMP4, mirroring territory pattern formation during embryonic development, including olfactory territory specification (Aguillon et al., 2016). These parallels suggest that mechanical forces may similarly govern OE folding and territorial organization, presenting an exciting direction for future investigation.

## 4 Conclusion

The OE emerges as a powerful model system to unravel how mechanical forces, architectural organization, and molecular programs collectively regulate stem cell plasticity and tissue morphogenesis. While other systems have revealed that forces can direct fate decisions, enable plasticity, and drive morphogenesis, the OE uniquely integrates these mechanisms within a sensory pseudostratified epithelium. The basal localization of HBCs, the lamellar folding architecture, and the conserved mechanosensitive pathways (e.g., CXCR4, YAP, integrins) suggest that mechanical cues may orchestrate plasticity and morphogenesis across developmental and regenerative contexts. To advance this field, a toolkit of mechanobiological approaches is now available to dissect these interactions (Table1: Tools to study mechanobiology). Moving forward, three critical questions demand exploration: (1) How do mechanical forces interact with molecular programs (*i.e.*, TP63, PRC2) to regulate stem/progenitor cells plasticity? (2) What role does lamellar folding play in generating spatial heterogeneity and functional specialization? (3) Can the architectural principles of the OE inform regenerative strategies for other stratified epithelia? By leveraging single-cell analyses, *in vivo* force sensing, and comparative approaches, the OE offers an unparalleled opportunity to decode the mechanobiological grammar underlying tissue formation and regeneration.
